# Eastern Caribbean Circulation and Island Mass Effect on St. Croix, US Virgin Islands: A Mechanism for Relatively Consistent Recruitment Patterns

**DOI:** 10.1371/journal.pone.0150409

**Published:** 2016-03-04

**Authors:** Laurent Marcel Chérubin, Lysel Garavelli

**Affiliations:** Florida Atlantic University, Harbor Branch Oceanographic Institute, Fort Pierce, Florida, United States of America; Fisheries and Oceans Canada, CANADA

## Abstract

The northeastern Caribbean Sea is under the seasonal influence of the Trade Winds but also of the Orinoco/Amazon freshwater plume. The latter is responsible for intensification of the Caribbean Current in general and of its eddy activity in the northern part of the Caribbean Sea. More importantly, we show in this study that the front of the freshwater plume drives a northward flow that impinges directly on the island of St. Croix in the United States Virgin Islands. The angle of incidence of the incoming flow controls the nature of the wake on both sides and ends of the island, which changes from cyclonic to anticylonic wake flow, with either attached or shed eddies. Using an off-line bio-physical model, we simulated the dispersal and recruitment of an abundant Caribbean coral reef fish, the bluehead wrasse (*Thalassoma bifasciatum*) in the context of the wake flow variability around St. Croix. Our results revealed the role played by the consistent seasonal forcing of the wake flow on the recruitment patterns around the island at the interannual scale. The interannual variability of the timing of arrival and northward penetration of the plume instead controls the nature of the wake, hence the regional spatial recruitment patterns.

## Introduction

The Caribbean Sea is a conduit for the upper part of the northward-flowing meridional overturning circulation [1–3] and has two nearly equal sources of inflow water [3, 4]. North of 15°N near Martinique, Caribbean inflow is primarily fed by the southern branch of the Subtropical Gyre, which consists of Gulf Stream water returning southwestward in the North Equatorial Current. South of 15°N, Caribbean inflow is primarily of tropical and South Atlantic origin. South Atlantic water crosses the equator in the North Brazil Current (NBC) and flows northwestward along the continental margin of South America in the form of a coastal current [5] and NBC rings [6–9], as well as some Ekman transport in the ocean interior [10].

Sea-surface salinity maps of the eastern Caribbean and Sea-viewing Wide Field of view Sensor (SeaWiFS) color images show a seasonal freshwater plume from the Orinoco and Amazon Rivers extending northwestward across the Caribbean basin from August to November, which is three to four months after the peak of the seasonal rains in northeastern South America [11, 12]. The plume enters the Caribbean Sea by two main inflows driven by the NBC and its rings. The southern inflow, enters the Caribbean Sea south of Grenada Island (near 11°N) and becomes the main branch of the Caribbean Current in the southern Caribbean, and the northern inflow passes northward around the Grenadine Islands and St. Vincent (near 14°N). As NBC rings stall and decay east of the Lesser Antilles, they release riverine water into the northern part of the eastern Caribbean Sea merging with inflow from the North Equatorial Current.

Using drifter data, Chérubin and Richardson [12] showed that three to four times more anticyclonic eddies are formed during the period August-December and that the Caribbean Current is seasonally intensified near 14°N, partly by the inflow of the river plumes. The low-salinity plume coincides with a negative potential vorticity anomaly, which sustains an anticylonic circulation in a northeastern Caribbean Sea, hence a northwestward flow on the western front of the plume as it enters the Caribbean Sea [12]. Therefore, the large-scale drivers of the northeastern Caribbean Sea circulation are responsible for its interannual seasonality, which makes it a consistent seasonal circulation pattern through the years.

**Fig 1:**
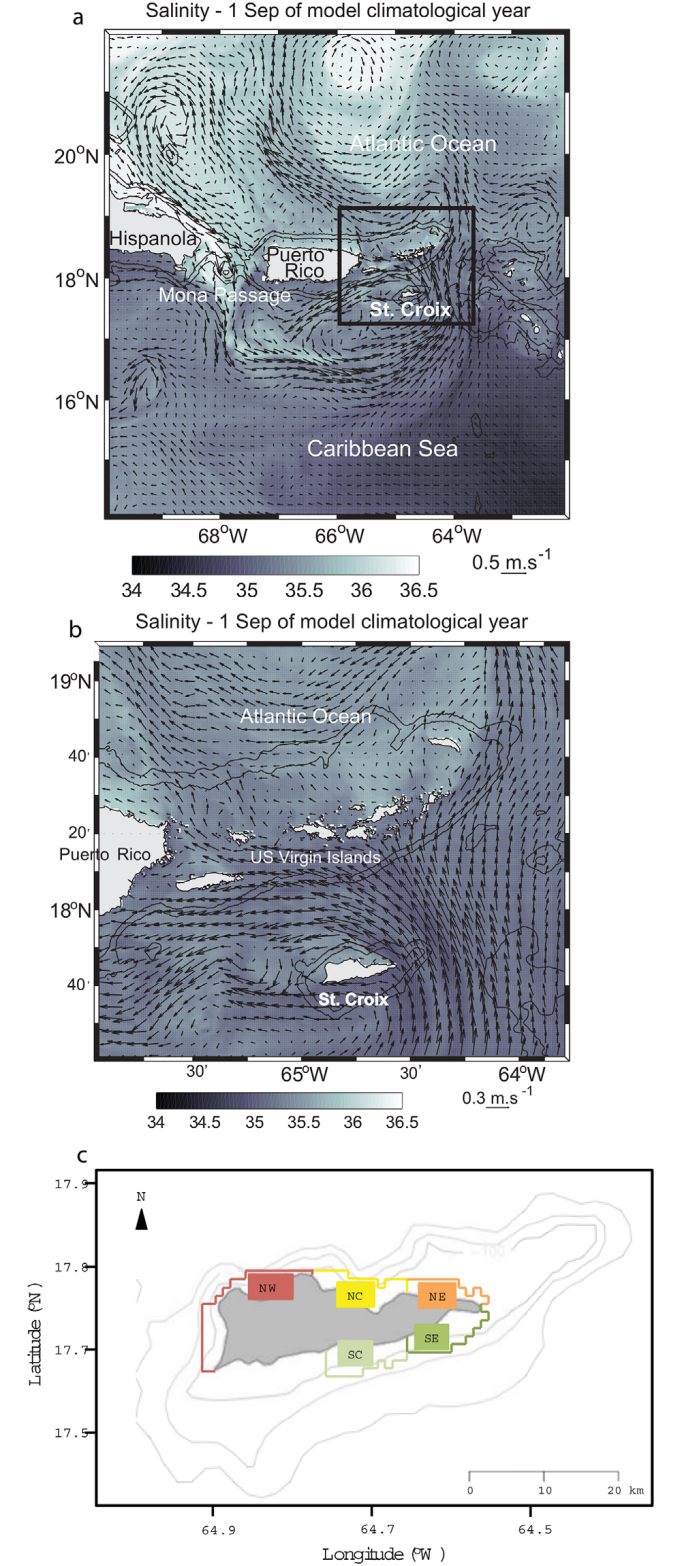
Snapshot of the flow field vectors overlaid on salinity in two of the model grids. (a) Parent grid, which encompasses the northeastern Caribbean Sea. The black square shows the contour of the first child grid. (b) First child grid. (c) Habitat polygons corresponding to both virtual release and recruitment areas around St. Croix.

The island of St. Croix, United States Virgin Islands, is located in the northeastern Caribbean ~70 km south of the Puerto Rico-Virgin Islands shelf, from which it is separated by a deep (>4000 m) trench, and 180 km from the nearest island to the east (Fig 1). This relative isolation confers an island-specific oceanography to St. Croix, which under the direct influence of the Trade Winds and also of the intense seasonal eddy activity that is associated with the arrival of Orinoco/Amazon freshwater plume. Caselle and Warner [13] studied the recruitment patterns around St. Croix of an abundant Caribbean coral-reef fish, the bluehead wrasse (*Thalassoma bifasciatum*), and found that recruitment increased from east to west on the north shore and from west to east on the south shore. Hamilton et al. [14] showed that this pattern was consistent at the monthly and annual scale. At a seasonal scale, higher recruitment was observed from June through August on the north shore and in September on the south shore, particularly at the eastern site. Harlan et al. [15] suggested that the recruitment patterns observed on St. Croix were produced by the formation and evolution of a convergence eddy in St. Croix’s wake. Using high frequency (HF) radar-derived surface current measurements at the northwestern corner (Butler Bay) of St. Croix, Harlan et al. [15] showed that the island wake flow exhibited eddy-like features and flow reversals. Wake regions are known for conditions that foster retention close to islands, increasing chances of both successful recruitment and larval abundance [16, 17].

In this study we aim to understand the interannual consistency of the spatial patterns of recruitment of *T*. *bifasciatum* observed by Caselle and Warner [13] and Hamilton et al. [14]. We believe that the consistency of the large-scale flow drives the consistency of the wake flow through the years, which in turn shapes the spatial recruitment pattern levels around the island of St. Croix. To begin we use ocean color data and a high-resolution, multi-year simulation of the northeastern Caribbean Sea circulation [12, 18, 19] to characterize the large scale flow consistency through its seasonality and then the subsequent seasonal variability of the island wake of St. Croix, which successfully reproduces the island-scale flow features observed by Harlan et al. [15]. Using a biophysical model coupled to our ocean model, we are able to reproduce the observed spatial recruitment patterns of *T*. *bifasciatum* and their consistency, which we show is directly related to the large scale incoming flow seasonality.

## Materials and Methods

### The Regional Oceanic Modeling System

The multiyear numerical simulations used in this study were produced with ROMS-AGRIF [20, 21], which performs local refinement via nested grids and has the ability to manage embedded levels and to do solution-adaptive grid refinement. Our model consisted of three embedded simulations, each on a nested grids run in a sequential mode, allowing one-way forcing from the parent to the child model at each time step of the former.

The model domain of the parent grid was the northeastern Caribbean (Fig 1a) encompassing some of the Windward Islands, the US and British Virgin Islands, Puerto-Rico, and part of Hispañola (14–23°N, 71–61°W). The first nested grid horizontal resolution was 1 km and encompassed the US and British Virgin Islands (Fig 1b), and the second nest horizontal resolution was 330 m and encompassed primarily St. Thomas (not shown). In all three grids the water column was discretized with 25 layers using the terrain-following s-coordinate. Two topographic datasets were used: (1) for the parent grid and the first child grid, the Global Topography dataset with a horizontal grid spacing of 1 km (GTOPO 30, http://edc.usgs.gov/products/elevation/gtopo30/gtopo30.html), and (2) for the second child grid, the National Geophysical Data Center Coastal Relief Model (http://www.ngdc.noaa.gov/mgg/coastal/grddas09/grddas09.htm), which has a 90 m resolution. Two types of simulations were used. For the climatological simulation, numerical model variables of the ocean state (temperature and salinity) were relaxed monthly to the World Ocean Atlas climatology (http://www.nodc.noaa.gov/OC5/WOA05/pr_woa05.html) at the open-ocean boundaries and for realistic years 2007–2009 they were relaxed weekly to the Hybrid Coordinate Ocean Model global ocean predictions [22]. The climatological simulation was forced at the ocean surface by monthly winds, heat, and water fluxes from the Comprehensive Ocean-Atmosphere Dataset. The realistic simulations were forced by three-hourly wind, air temperature, relative humidity, evaporation and precipitation rates obtained from North American Regional Reanalysis. Wind stress was calculated by the model’s air-sea fluxes bulk formulation. Four-hourly net surface shortwave and longwave heat fluxes and net shortwave radiation were obtained from the National Centers for Environmental Prediction (NCEP)/National Center for Atmospheric Research (NCAR) reanalysis. The model sea surface temperature (SST) and salinity (SSS) were relaxed daily to, respectively, the coarser 4 km resolution night SST from the National Oceanic and Atmospheric Administration’s Advanced Very High Resolution Radiometer Pathfinder SST v5 and to monthly SSS from the NCEP Global Ocean Data Assimilation System. The model was spun-up for six months, after which tides were forced at the boundary with the TPXO6 global tide model [23] using six tidal components: M2 (principal lunar, semi diurnal), S2 (principal solar, semi-diurnal), N2 (larger lunar elliptic, semi-diurnal), K2 (luni-solar, semi-diurnal), K1 (luni-solar, diurnal), and O1 (principal lunar diurnal).

This climatological simulation has been used to study the transport of Elkhorn coral (*Acropora palmata*) larvae across Mona Passage [18], the influence of Orinoco/Amazon riverine water on the variability and eddy field of the Caribbean Current [12] and the flow pathways at a spawning aggregation site located at the shelf break south of St. Thomas [19].

### Larval transport model

The larval dispersal of *T*. *bifasciatum* in St. Croix was modeled using the individual-based offline Lagrangian tool Ichthyop v. 3.2 [24]. The algorithm is based on a forward Euler advection scheme using velocity fields from ROMS. The latitude, longitude and depth of each virtual egg and larva were recorded every hour in three dimensions. Horizontal diffusion was included in the model (with a turbulent dissipation rate є = 10–9 m².s-3; see Peliz et al. [25] for details). Five release and settlement areas were selected along the coast of the island (Fig 1c). Three were located on the northern (leeeward) shore: northwest (NW), central north (NC), and northeast (NE); and two on the southern (windward) shore: southeast (SE) and central south (SC). Because the shelf on the northwestern shore is narrower than on the south eastern shore, the maximum depth of the release and settlement zones is larger there (400 m for NW, 250 m for NC, and 100 m for NE versus 150 m for SE and SC). Because the recruitment periods of *T*. *bifasciatum* are the summer and the beginning of autumn [13, 14] and the planktonic larval duration lasts 38–78 days [26, 27], we selected 22 April to 22 August as the spawning period. Spawning was simulated by the release of 5000 eggs every day in all areas for the climatological simulation and model years 2007 to 2009. Spawning depth was between 0 m and 20 m, and the eggs were initially transported as passive particles for 48 hours. After the initial passive advection period in the upper 20 m, particles (i.e., virtual larvae) were stochastically moved vertically with a five-day time step, following a probability density function distributed over the 10 upper layers (1 m, 5 m, 10 m, 20 m, 30 m, 40 m, 50 m, 55 m, 60 m, 80 m) based on observed vertical distribution of developmental stages of larvae in the field [28, 29]. The planktonic larval duration was set between 38 days (end of pre-competency period for *T*. *bisfasciatum*) and 78 days (maximum period for metamorphosis [26, 27]). Finally, to account for decreased survivorship with extended planktonic duration [30], a daily mortality rate of 0.1 was applied [31]. To date, there are no mortality estimates for the target species. The virtual larvae were considered as recruited when they were competent to settle and located in settlement areas.

We represented the results of the simulations as connectivity matrices. Each value of the connectivity matrix C_i,j_ is called transport success and is calculated as the percentage of larvae released from zone *j* that settled to zone *i*. We also analyzed the monthly settlement of larvae in our simulations for NW (i.e., the western site on the northern shore) and SE (i.e., the eastern site on the southern shore). For this, we first calculated the proportion of larvae successfully transported to a destination site and then normalized this proportion by the maximum number of larvae transported among all release sites. We compared our results with monthly recruitment observed in St. Croix for *T*. *bifasciatum* obtained during field studies by Caselle and Warner [13] and Hamilton et al. [14].

## Results

### Large-scale flow in the eastern Caribbean

In the northeastern Caribbean Sea, the August-September period bears the influence of the fresh water plume from the Orinoco and Amazon rivers, as observed by Corredor and Morell [32] using a 10-year time series of *in situ* observation from the Caribbean Time Series station south of Puerto-Rico. Hu et al. [11], tracking this flow in the eastern Caribbean using SeaWiFS ocean color time series of colored dissolved organic matter calibrated by Salinity Profiling Autonomous Lagrangian Current Explorer floats, showed that the salinity anomaly of the plume enter the northeastern Caribbean Sea every year starting in June/July and propagates to the northwest. Chérubin and Richardson [12] showed that the fresh water anomaly creates an anticyclonic circulation. The western front of the plume is often populated with cyclones, and this eddy field drifts westward in the Caribbean Sea [12] such that the current in the frontal area is northward-moving south of St. Croix.

**Fig 2:**
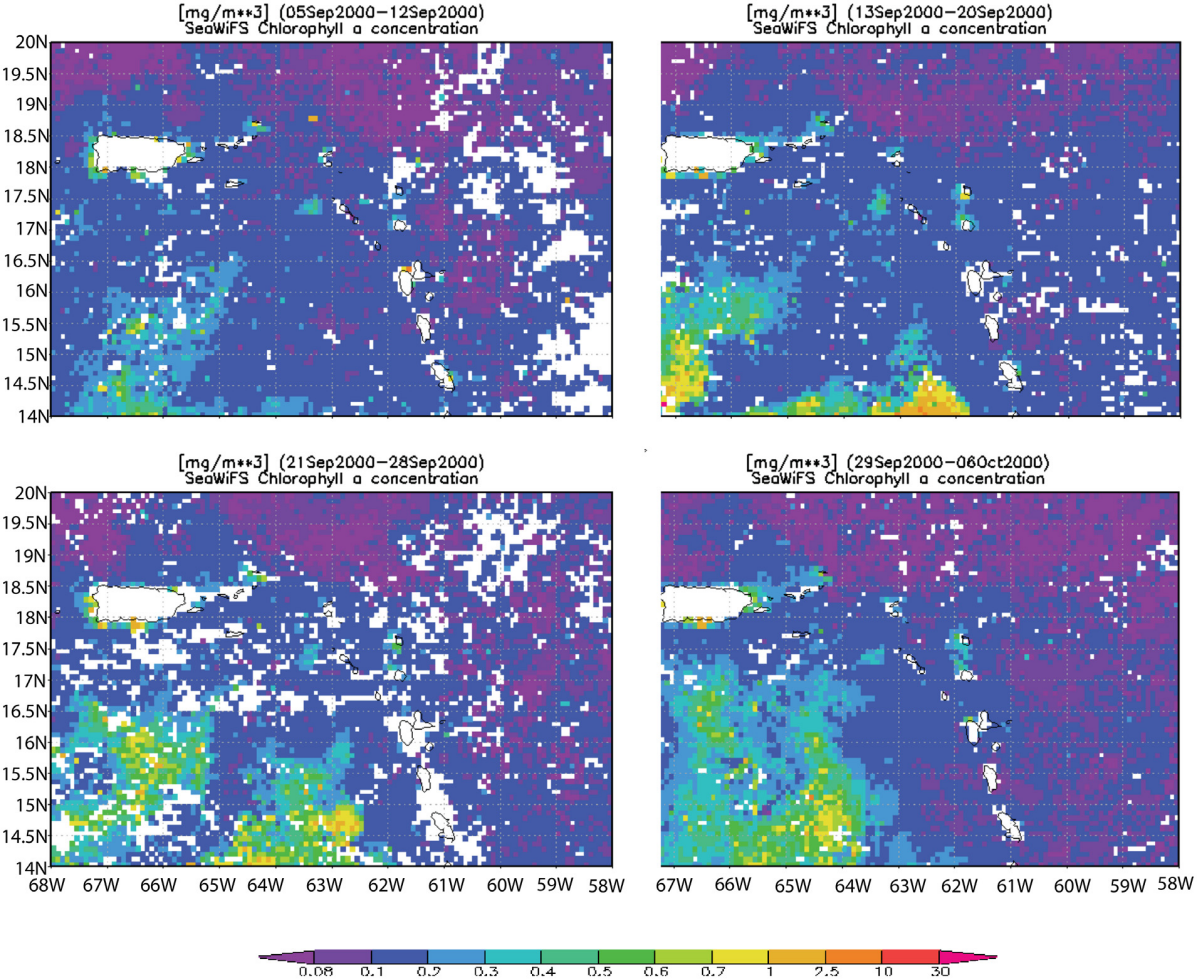
Time-series of 8-day average SeaWiFS chlorophyll a concentration (mg. m^-3^) images from 05 September to 06 October 2000 showing the northward advection of the Chl-a plume.

**Fig 3:**
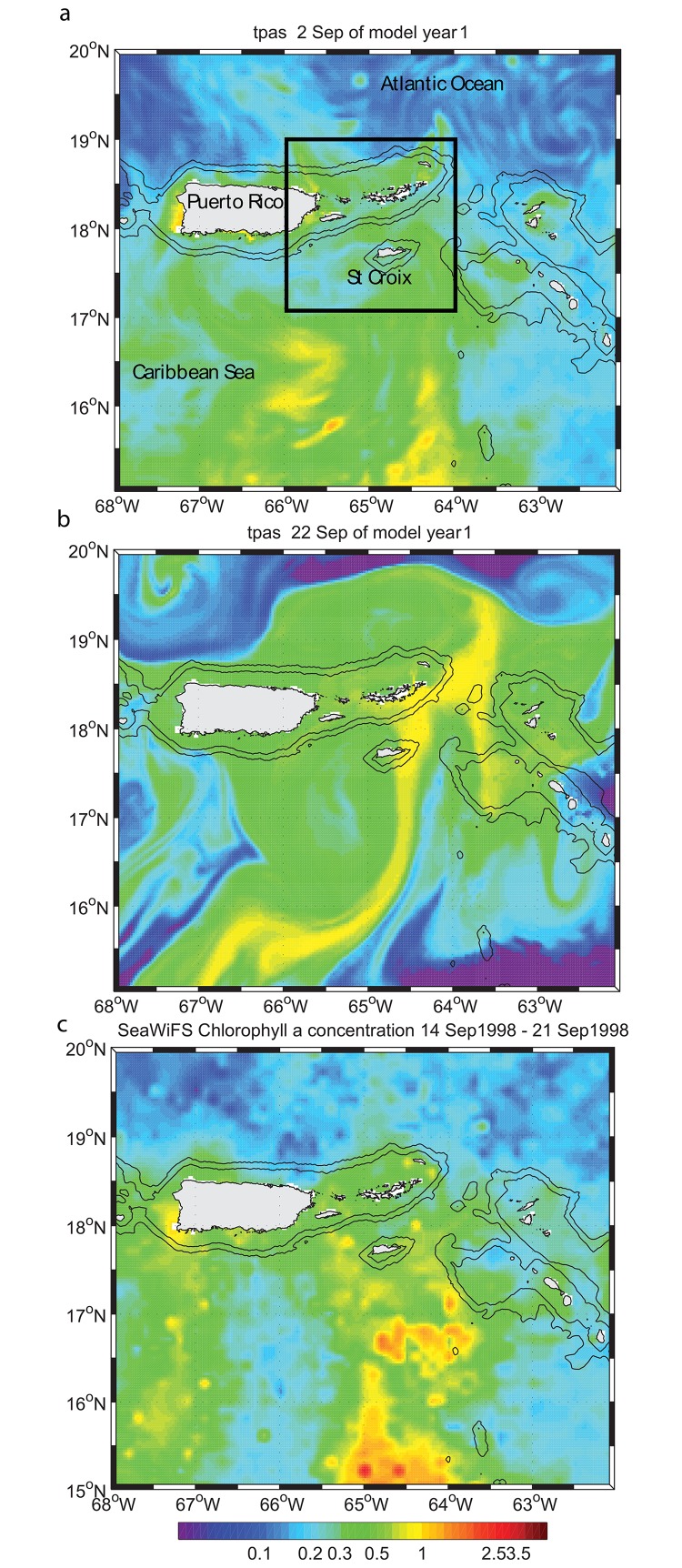
Chlorophyll a concentration in model parent grid in mg.m^-3^. (a) Model initial field obtained from SeaWiFS chlorophyll a concentration 8-day average using the time period 29 September 2000–06 October 2000. Black square shows the boundary of the first child grid. (b) Model field twenty-two days after initialization. (c) same as (a) for the time period 14–21 September 1998.

The presence of a northward current in September is shown by the northward drift of the chlorophyll *a* (*Chl*-*a*) plume in SeaWiFS images in Fig 2, which shows weekly averages between 5 September and 6 October 2000. To confirm the presence of this northward transport in the model, the latter was initialized with the eight-day average SeaWiFS *Chl*-*a* concentration for the time period 29 September to 06 October 2000, as the *Chl-a* plume enters the model domain. The SeaWiFS image was converted into a passive tracer concentration and used to represent initial conditions in the simulation run on 2 September model time (Fig 3a). Within 20 days the tracer had reached the southern shores of St. Croix (Fig 3b) and beyond. In this tracer experiment we did not simulate the biological cycle of *Chl*-*a*, which explains why the model passive tracer was transported beyond the limits observed in the SeaWiFS climatology. Nonetheless, such northward transport was observed during 14–21 September 1998 (Fig 3c), which substantiates the model dynamics. The drift time-scale between 15°N and 18°N was approximately one week, as suggested by the *Chl*-*a* imagery. Characterization of the plume’s evolution has been similar in multiple years [11, 32], suggesting consistency in the presence of this current to the south of St Croix at the beginning of the wet season. We can now assume that the incoming flow on St. Croix during this time period is mostly northward and we will show in the following that only such flow direction can explain the wake observed by Harlan et al. [15].

### St. Croix wake flow features

#### Model wake flow and observations

**Fig 4:**
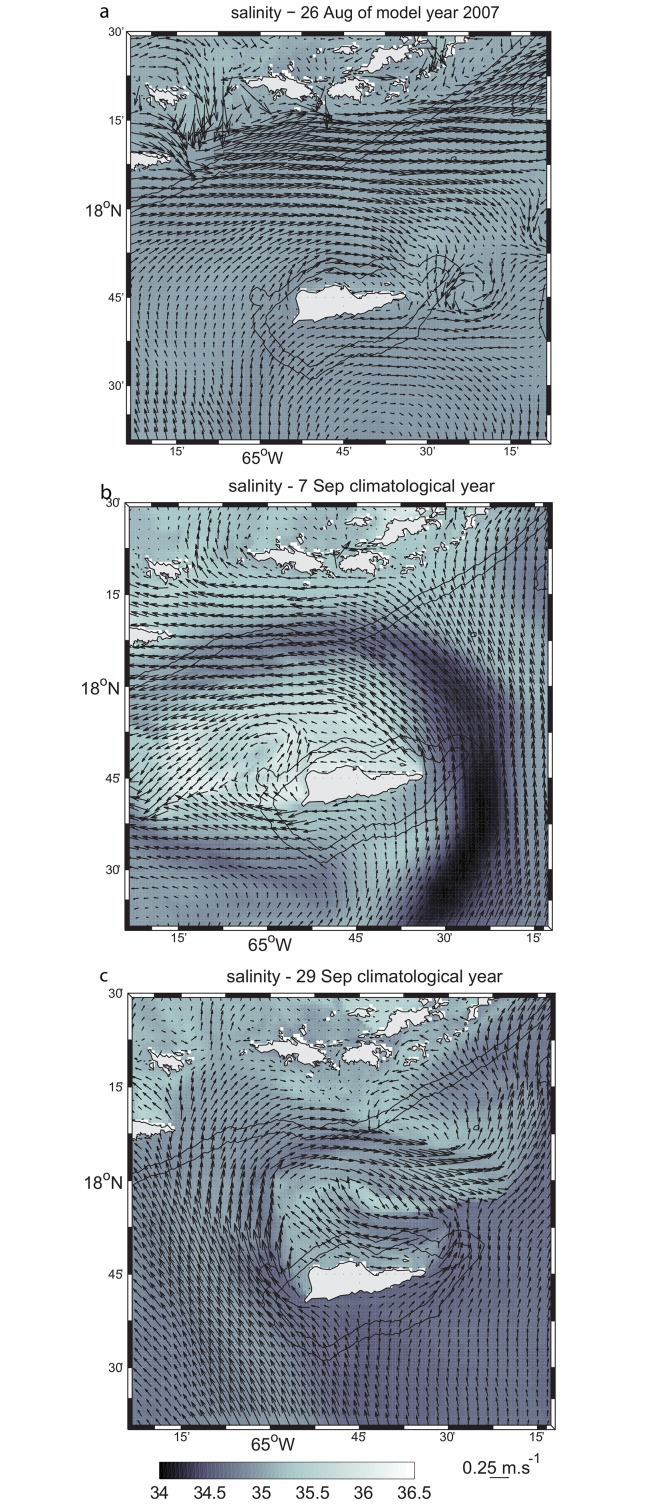
Surface flow field snapshots from the model first child. (a) Snapshot obtained on 26 August of model year 2007. (b) Flow field snapshot on 7 September of the climatological year. (c) Same as (b) on 29 September.

In order to study the evolution of possible convergence or eddy formation in the wake of St. Croix, a relatively small region (10–100 km^2^) northwest of the island, Harlan et al. [15] examined the two-dimensional surface circulation using HF radar-derived surface currents. The radar data were collected from 10 June 1997 until 2 September 1997 at hourly intervals. The typical flow pattern during much of the experiment’s duration consisted of a northwestward flow (280–300°) with an approximate mean speed of 0.2 ms^-1^ throughout most of the radar coverage area, but with a smaller near-shore region off the northwestern tip of the island exhibiting weaker transient sub-mesoscale features. These current patterns snapshots shown in Harlan et al.’s [15] Figs 4, 3 and 6 respectively visually match the sub-mesoscale features in the model shown in Fig 4a–4c.

The model snapshots show that the wake features north of St Croix are created by the incoming northward flow south of St. Croix; when incoming from the southwest, wake eddies form at the eastern point on St. Croix (Fig 4a). When the incoming flow is deflected to the east, it wraps around the island and flows to the west on the north side (Fig 4b); when deflected to the west, it wraps around the western side of St. Croix and flows to the east (Fig 4c). In Fig 4a, the wake zone can extend east of the island for more than 70 km (not shown). In Fig 4b, the wake flow extends for only 30 km and less than 10 km north of St. Croix. In Fig 4c, the northern wake appears to be more extended—up to 30 km from St. Croix. In the latter, the sub-mesoscale meander of the deflected eastward branch of the incoming current seems to produce meanders along the southern shelf of the Virgin Islands (Fig 4c), suggesting even further influence of St. Croix’s wake flow.

In general, conditions required to form the coherent and steady vortex structures in the wake of St. Croix are not present. In particular the flow is not symmetrical on both sides of the island most of the time (Figs 1 and 4) and cannot reconnect downstream of the island as it is blocked by the shelf of St. Thomas (Fig 1; [33]). Moreover, the flow could be part of a mesoscale eddy, which could help curve the flow westward as it passes by St. Croix (Fig 1a), breaking the wake theory assumptions of rectilinear incident uniform current.

#### Cyclone-dominated wake flow

**Fig 5:**
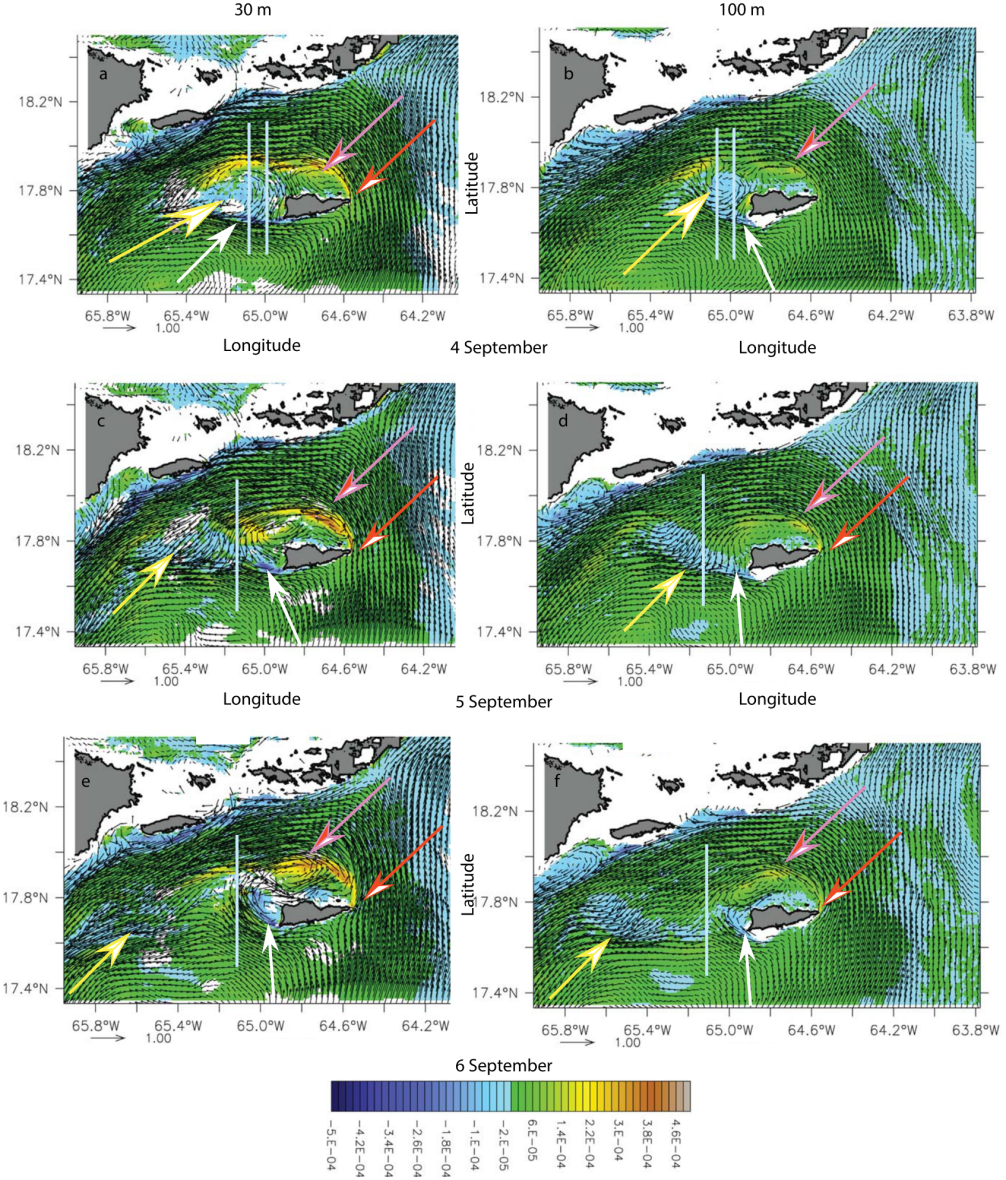
Potential vorticity anomaly (PVA) (s^-1^) time series at 30-m (left column) and 100-m (right column) during the cyclonic wake-flow period in model month September of the climatological simulation, for model day 4 (a, b), 5 (c, d) and 6 (e, f). White (orange) arrows show the negative (positive) vorticity anomaly generation region. Yellow (pink) arrows indicate the anticyclone (cyclone). Grey lines show the location of the cross sections in Fig 6.

As previously shown, St. Croix’s wake dynamics changed with the angle of incidence of the flow on the islands. The first case we identified in the simulation forced by climatological atmospheric fluxes showed that St. Croix was embedded in a cyclonic eddy hugging the southern shelf of Puerto Rico and the Virgin Islands in model months August and September (Fig 5). Because of the current curvature, the wake region was west of St. Croix. However, the wake regime consisted of a quasi-stationary cyclonic eddy north of St. Croix (pink arrows) and a short-lived anticyclonic eddy (yellow arrows) west of the island. Downstream, the wake consisted of the passage of meanders associated with decaying anticyclones (Fig 5c and 5d, yellow arrows). The characteristic vertical integrated velocity was U_o_ = 0.7 m.s^-1^, the vertical extent of the flow H_o_ = 250-m, the Rossby radius R_d_ ~ 9-km, Ro = 0.15, and Bu = 0.55. Since the ROMS viscosity is implicit, we assume that Re is infinite. Based on Dong et al.’s [34] study of island wake in deep water, which assumes negligible bottom vorticity effects, a mesoscale anticyclonic eddy should exist with the cyclonic eddy in our simulation. The anticyclone was indeed present at the western end of St. Croix, although it was periodically destroyed by the wake (Fig 5c and 5d, day 5 September). According to Coutis and Middleton [35], however, small Ro should also favor attached wake eddies, which could explain the long-lived cyclone; it vanished after 28 days. Based on the shape of the island, which both ends could be seen as a promontory, the wake regimes could instead be driven by a cape effect, which is likely to generate only one type of vortices that could remain attached or shed from the cape [36].

**Fig 6:**
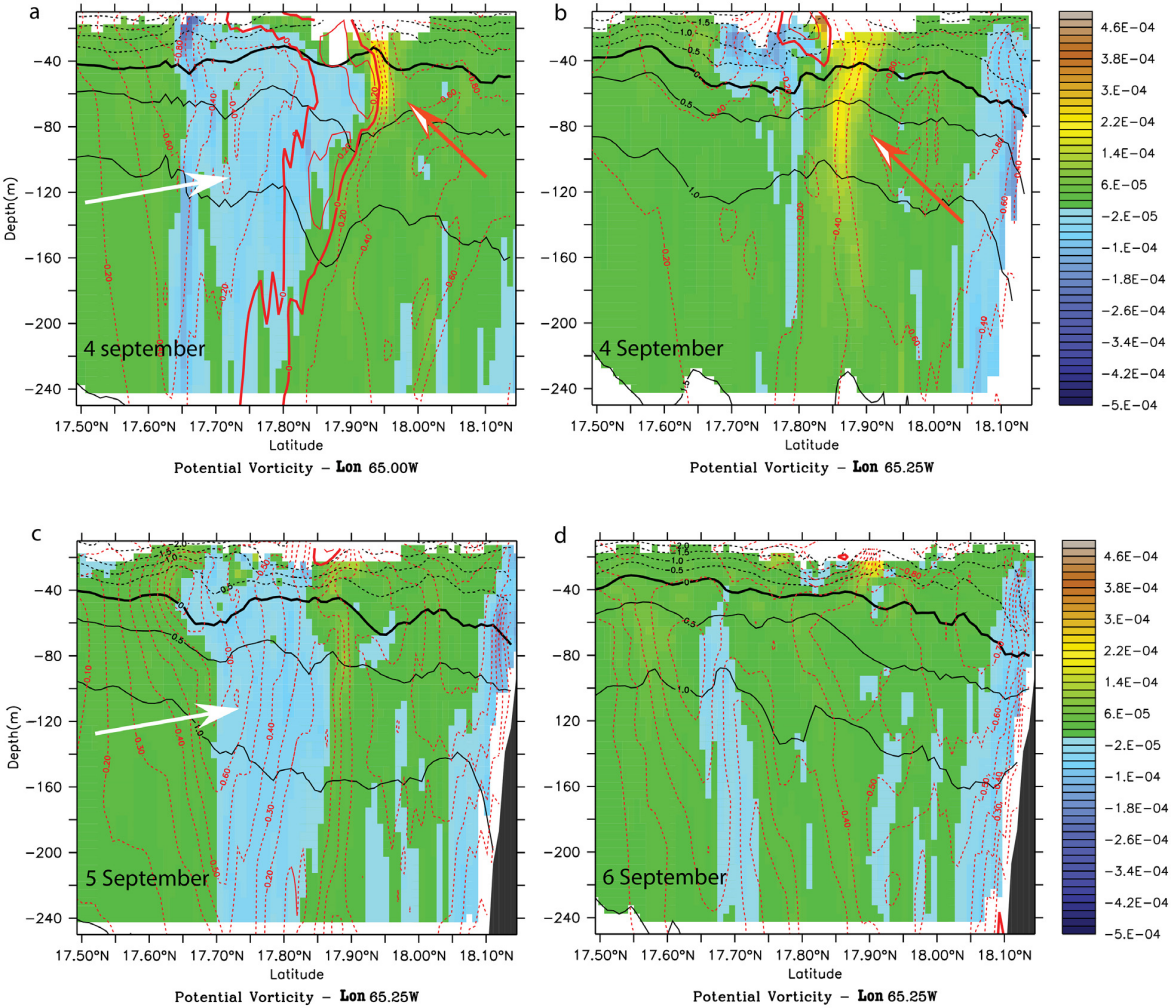
Potential vorticity anomaly (PVA) (s^-1^) meridional cross-sections during the cyclonic wake-flow period in model month September of the climatological simulation. (a) In the anticyclonic eddy at 65.00°W on model day 4 September. (b) In the wake of the anticyclonic eddy at 65.25°W on model day 4 September. (c) In the wake at 65.25°W on model day 5 September. (d) In the wake at 65.25°W on model day 6 September. White (orange) arrows indicate negative (positive) vorticity anomalies.

The presence of the cyclone on the north of St. Croix was associated with the generation of cyclonic vorticity (orange arrows) in the lee of the eastern tip of the islands and anticyclonic vorticity (white arrows) on the southwestern tip (Fig 5). Cyclonic vorticity (orange arrows) was surface-intensified whereas the negative vorticity (white arrows) generation was more barotropic (Fig 6a–6c). A potential vorticity anomaly section across the anticyclone on 4 September suggests a deeper generation of negative vorticity as the eddy extends below 250 m (Fig 6a) than for the cyclonic vorticity. North of the anticyclonic eddy, a positive vorticity filament originating from the island eastern tip (Fig 5) was present and was intensified down to the pycnocline and deeper, further away in the wake (Fig 6a and 6b). The anticyclonic eddy diameter was about 30 km, the length of St. Croix. On 5–6 September, although the negative vorticity remained, the eddy field had dissipated (Fig 6c) and the negative vorticity anomaly had advected away along with the surface cyclonic filament (Fig 6d). The deep vorticity maps show that the negative vorticity was generated in the lee of a deep southwestern promontory (Fig 5). Based on the work of Magaldi et al. [36] and the values of Ro, Bu, and Re_*f*_ in our simulation, the eddy regime should consist of eddy shedding, which is what we observed. Therefore, the cyclonic wake flow of St. Croix may consist of mixed island wake and cape eddies.

**Fig 7:**
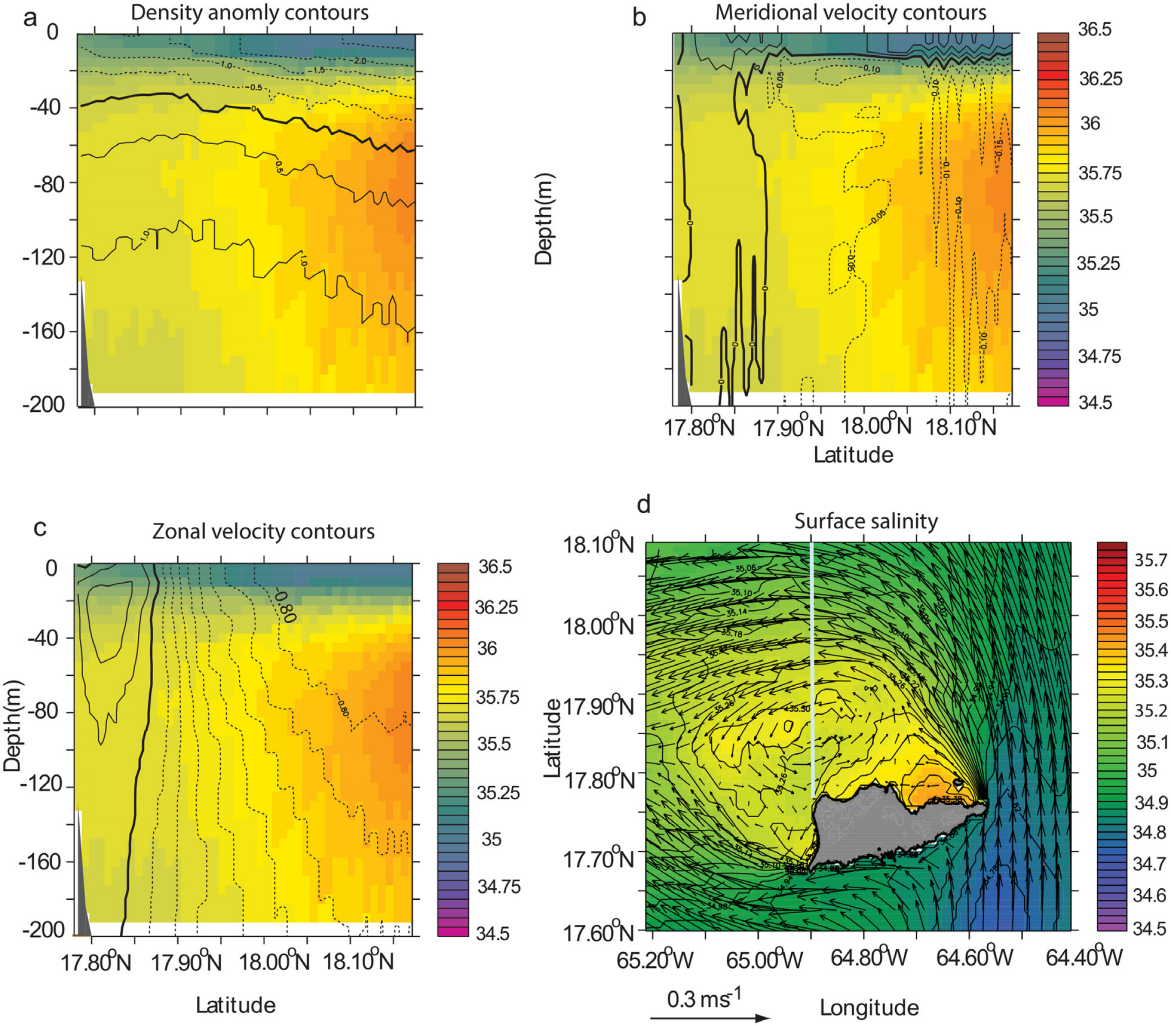
Mean salinity of the north side of St. Croix during model month 1–23 September of the climatological simulation. (a) salinity meridional cross section at 64.9°W (see (d)) overlaid with density anomaly contours. Thick line shows 0 contour. Contour interval is 0.5. Dashed (solid) lines show negative (positive) anomalies. The reference density is 1025 kg.m^-3^. (b) Same cross section as (a) overlaid with meridional velocity contours. Velocity increment is 0.05 m.s^-1^. (c) Same cross section as (a) with zonal velocity contours. Velocity increment is 0.1 m.s^-1^. (d) Mean horizontal surface salinity field (color) overlaid with mean velocity vectors. The grey line shows the location of the cross section.

The surface flow on the northern side of St. Croix in the model is characterized by higher salinities than its surrounding waters in some instances (Fig 4b). Waters masses present in the eastern Caribbean Sea originate from the western Atlantic Ocean, where tropical surface waters, characterized by temperatures higher than 27°C, are present in the first 50 m and salinity maximum waters (SMW), with salinities greater than 36.5 ppt, are found between 50 m and 100 m [32, 37]. The mean state of the vertical structure of the salinity in Figs 4b and 7a is typical of upwelling systems, where, in this particular case, saline water is upwelled at the coast and transported offshore by doming isopycnals close to the coast. The higher salinity in surface waters north of St. Croix could be explained by trade winds that blow parallel to the north coast throughout the year, driving upwelling along the coastline. The mean meridional circulation (Fig 7b) shows the offshore Ekman transport confined within the first 20 m of the upper water column, similar to the California upwelling system [38, 39]; deeper, a broad, slow, shoreward return flow is present. In addition to the wind-driven upwelling, the mean horizontal flow exhibits a cyclonic circulation (Fig 7c), which is not typical of island wake flow in deep water. Instead, in stratified water, one may expect the formation of dipoles that shed periodically [34]. Furthermore, frontal features driven by converging flows and upwelling-driven haline fronts are present along the northeast side of the wake and originate from St. Croix’s eastern end (Fig 7d). On the west end the frontal features are more diffused.

#### Anticyclone-dominated wake flow

**Fig 8:**
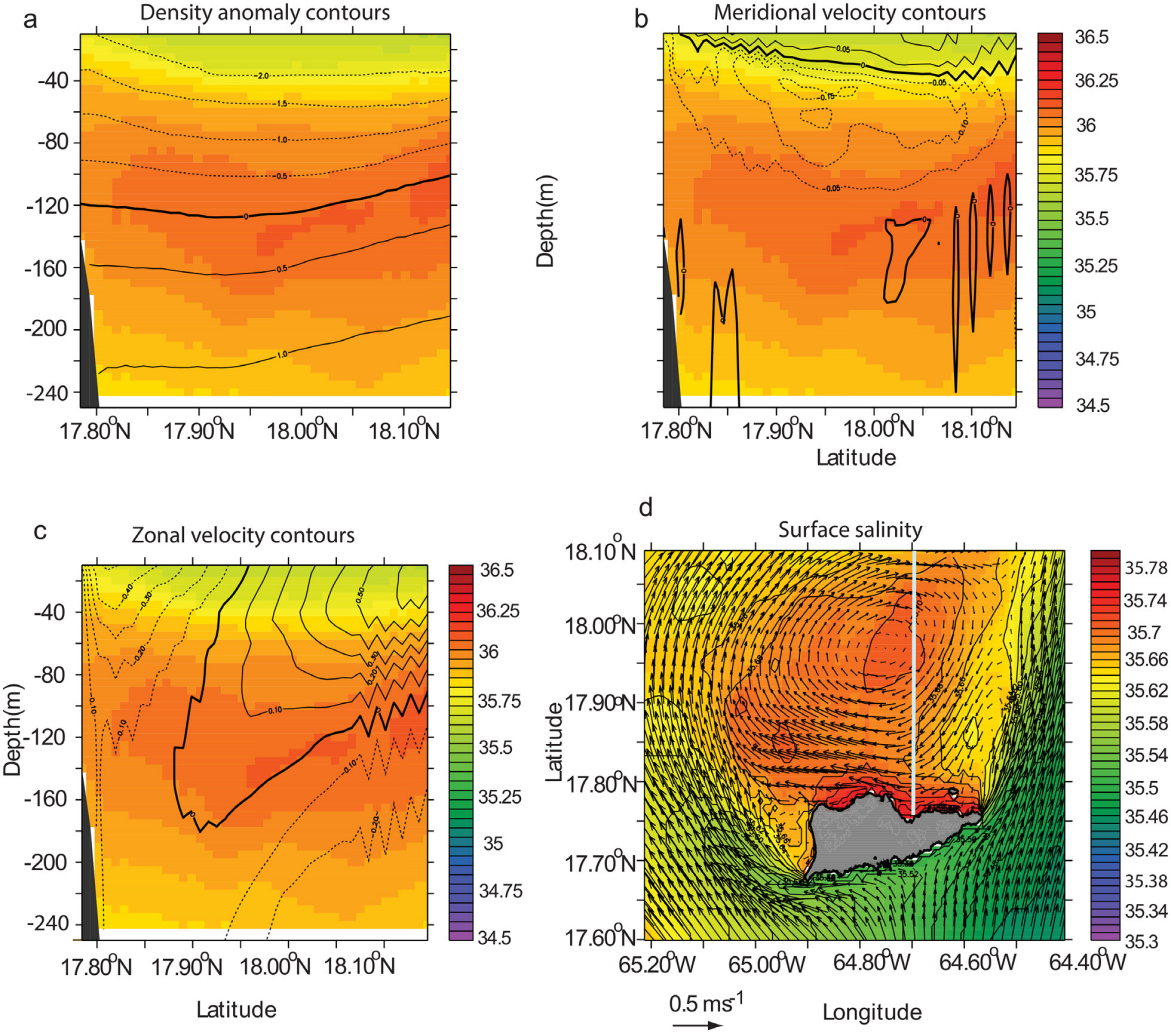
Mean salinity of the north side of St. Croix during model month 1–20 August 2007 of the real time simulation. (a) salinity meridional cross section at 64.7°N overlaid with density anomaly contours. Thick line shows 0 contour. Contour interval is 0.5. Dash (solid) lines show negative (positive) anomalies. The reference density is 1025 kg.m^-3^. (b) Same cross section as (a) overlaid with meridional velocity contours. Velocity increment is 0.05 m.s^-1^. (c) Same cross section as (a) with zonal velocity contours. Velocity increment is 0.1 m.s^-1^. (d) Mean horizontal surface salinity field (color) overlaid with mean velocity vectors. The grey line shows the location of the cross section.

In the presence of northeastward flow or eastward-deflected northward flow (Fig 4c), the mean circulation can be anticyclonic, as shown by Fig 8. This mean state was obtained in model year 2007. The anticyclonic circulation appeared to be coupled with the upwelling on the north side of St. Croix because isopycnals and isohaline rose at the coast (Fig 8a). The meridional velocity section is also typical of an upwelling system, with surface current directed away from the coast between 0 m and 40 m and the bottom flow directed toward the coast (Fig 8b). The zonal velocity confirms the anticyclonic circulation of the mean flow and is evident in the tilted axis of the anticyclone, which extends down to 160 m and is closer to the coast there than at the surface (Fig 8c). This tilt could be explained by the upwelling-induced transport toward the coast as shown by Fig 8b. Frontal features are also present north of the island (Fig 8d).

**Fig 9:**
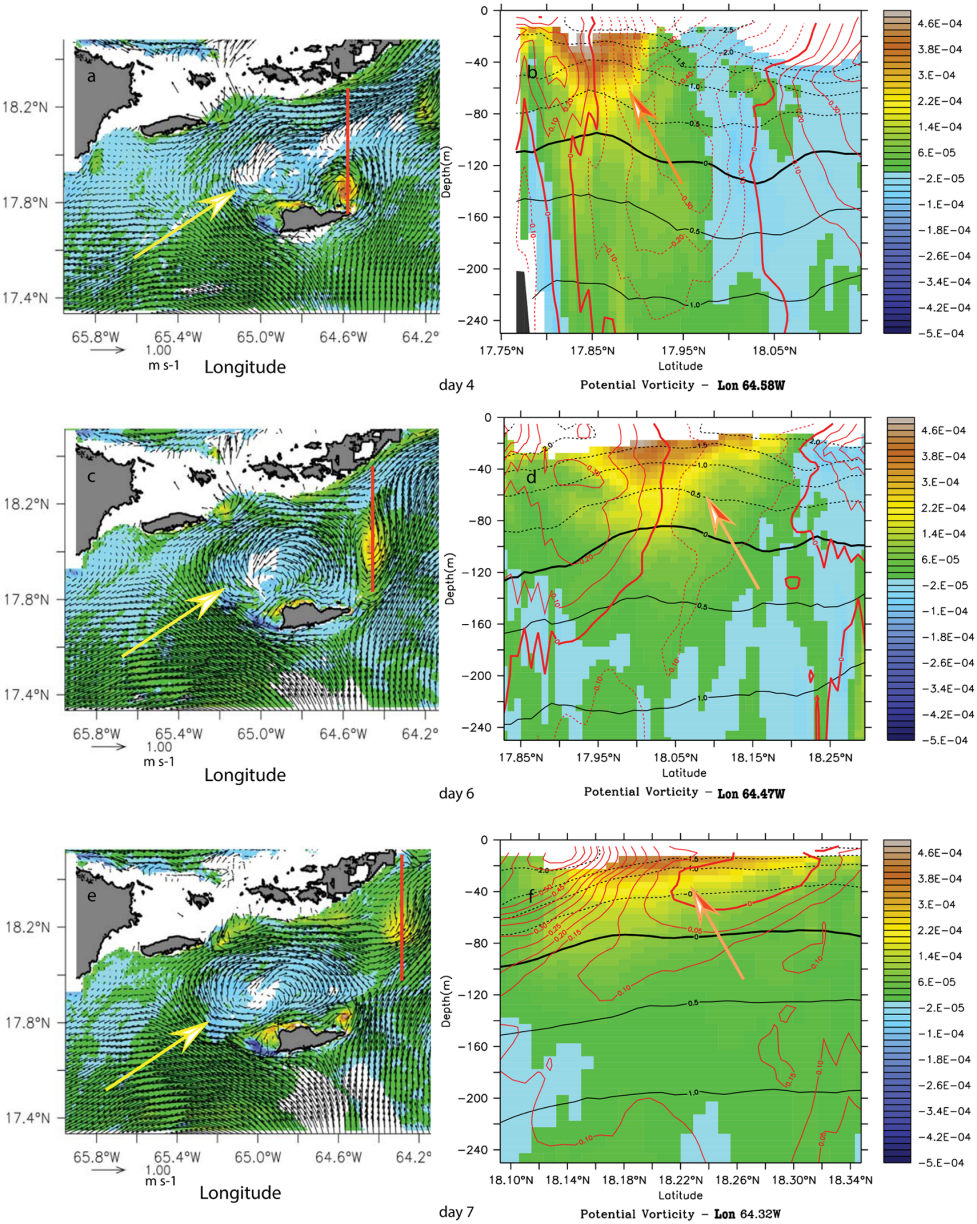
Potential vorticity anomaly (PVA) (s^-1^) snapshot at 30-m (left column) and meridional cross-section (right column) during the anticyclonic wake flow in August of model year 2007. (a) PVA on 4 August. (b) PVA meridional section in the cyclonic eddy at 64.58°W. (c) PVA on 6 August. (d) PVA meridional section in the cyclonic vorticity core at 64.47°W. (e) PVA on 7 August. (b) PVA meridional section in the cyclonic vorticity core at 64.32°W. Yellow arrows show the anticyclone and the red line shows the cross-section location across the cyclone. The orange arrow in the right panels shows the cyclonic vorticity.

In the second type of incident flow, which occurred in model month August 2007, the current was split between two branches flowing in opposite directions parallel to the southern coast of St Croix (Fig 9). The incoming flow was less swift (~ 0.3 m.s^-1^), hence Ro, according to Dong et al. [34] and Coutis and Middleton [35], should favor attached eddies. This wake flow was characterized by a long-lived (20 days) anticyclone north of St. Croix (white arrow) and a transient cyclone to the northeast (Fig 9). Cyclonic vorticity was locally produced at the south side of the eastern tip of St. Croix and along its northwestern shore by the recirculation flow of the anticyclone. Negative vorticity was generated at the southwestern tip of St. Croix. The anticyclone remained attached to the island while transient cyclonic eddies were shedding from the eastern tip of the island; these were entrained and strained by the stronger anticyclone, became elliptic, and turned into a positive vorticity anomaly with incoherent features within two days and 40 km of where they were formed. Vertical vorticity sections across a cyclonic eddy showed first a surface-intensified eddy with a signature extending down to 100 m when still attached to St. Croix (Fig 9). As it moved away, the signature became as shallow as 50 m. The cyclone diameter was about half the length of St. Croix and the anticylone diameter was about twice as large.

**Fig 10:**
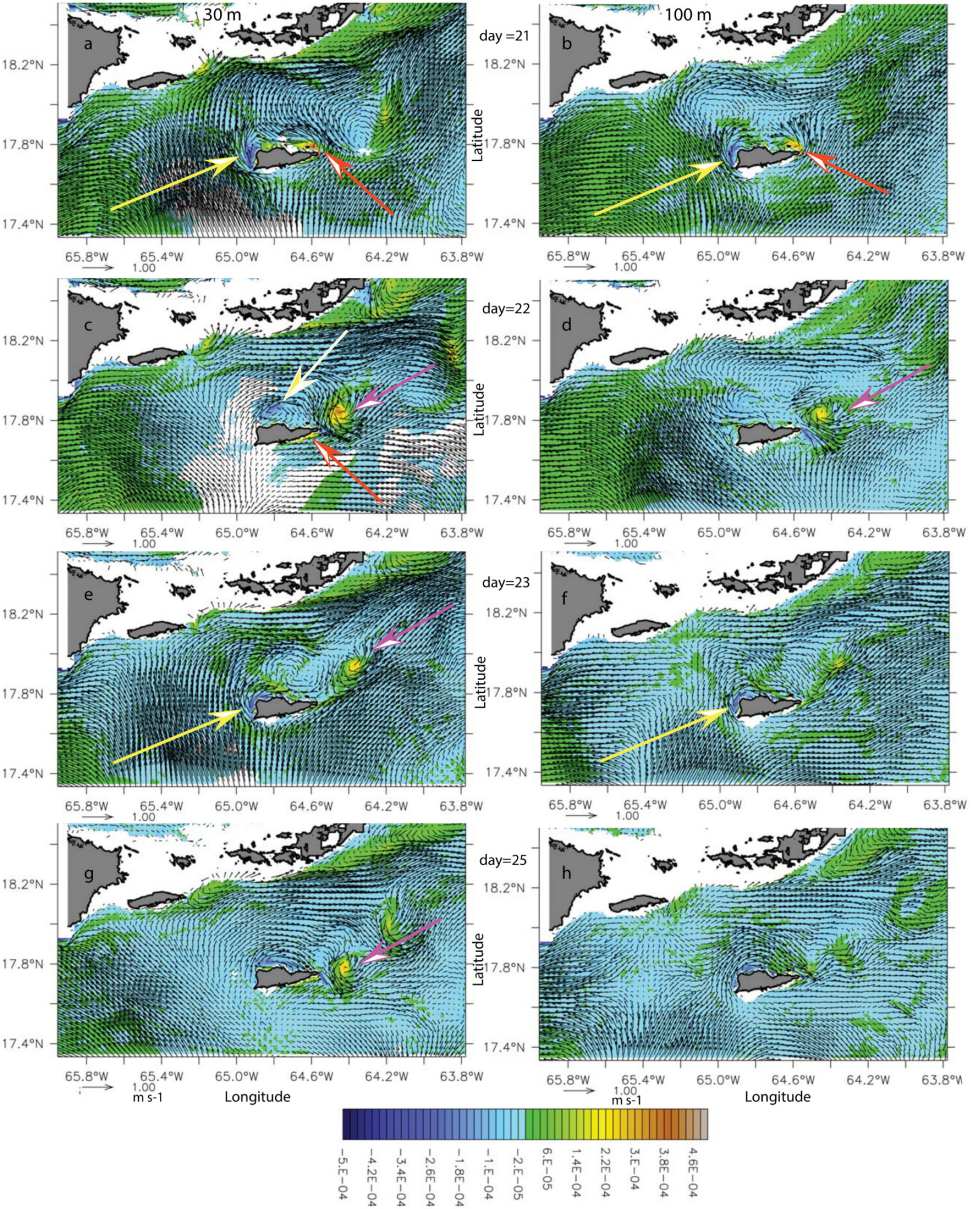
Potential vorticity anomaly (PVA) (s^-1^) time series at 30-m (left column) and 100-m (right column) during the eastern cyclones shedding wake-flow period in model month August 2007. From top to bottom, model day 21(a, b), 22(c, d), 23(e, f), and 25(g, h). Yellow (orange) arrows show the negative (positive) vorticity anomaly generation regions. White (pink) arrows show the anticyclone (cyclone).

As the incident current shifted to the northeast at the end of model month August, the current was eastward and parallel to the southern coast of St. Croix, and positive vorticity was generated on the eastern end of this coast (Fig 10, orange arrows). Because the incoming flow on the southwestern point of St. Croix was not forced above the southwestern deep cape, the negative vorticity generation was shifted to the western northwestern coasts (yellow arrows). The incoming flow was relatively weak (0.3–0.1 m.s^-1^), although it accelerated north of St. Croix. Subsequently, the anticyclonic eddy (yellow arrow on Fig 10c) became smaller—about the size of St. Croix—and intermittent. Similarly, the cyclones (pink arrows) became smaller (approximately 6 km radius; less than Rd) and were formed and ejected in less than two days at regular intervals from the eastern tip of St Croix, suggesting an eddy cannon; they were short-lived with no coherent features after two and a half days. In this case, the anticyclone had almost no deep signature (yellow arrow in Fig 10c), in contrast to the cyclone that was coupled with a deep (100-m) negative vorticity anomaly formed at the time of separation of the cyclone (Fig 10c, day 22).

The difference between the two anticyclonic wakes seems to be the influence of bottom topographic features (i.e., the subsurface cape to the southwest or the shelf platform to the southeast). Because of the enhanced friction on the bottom, the vorticity generation mechanism seems sustains stronger and larger eddies in the wake (Fig 9) than laterally generated vorticity, as shown by anticyclonic eddies in particular and cyclonic eddies that had a deeper and stronger vorticity signature when vorticity was generated on the southern shelf of St. Croix (Fig 10).

#### Other St. Croix wake flow regimes

**Fig 11:**
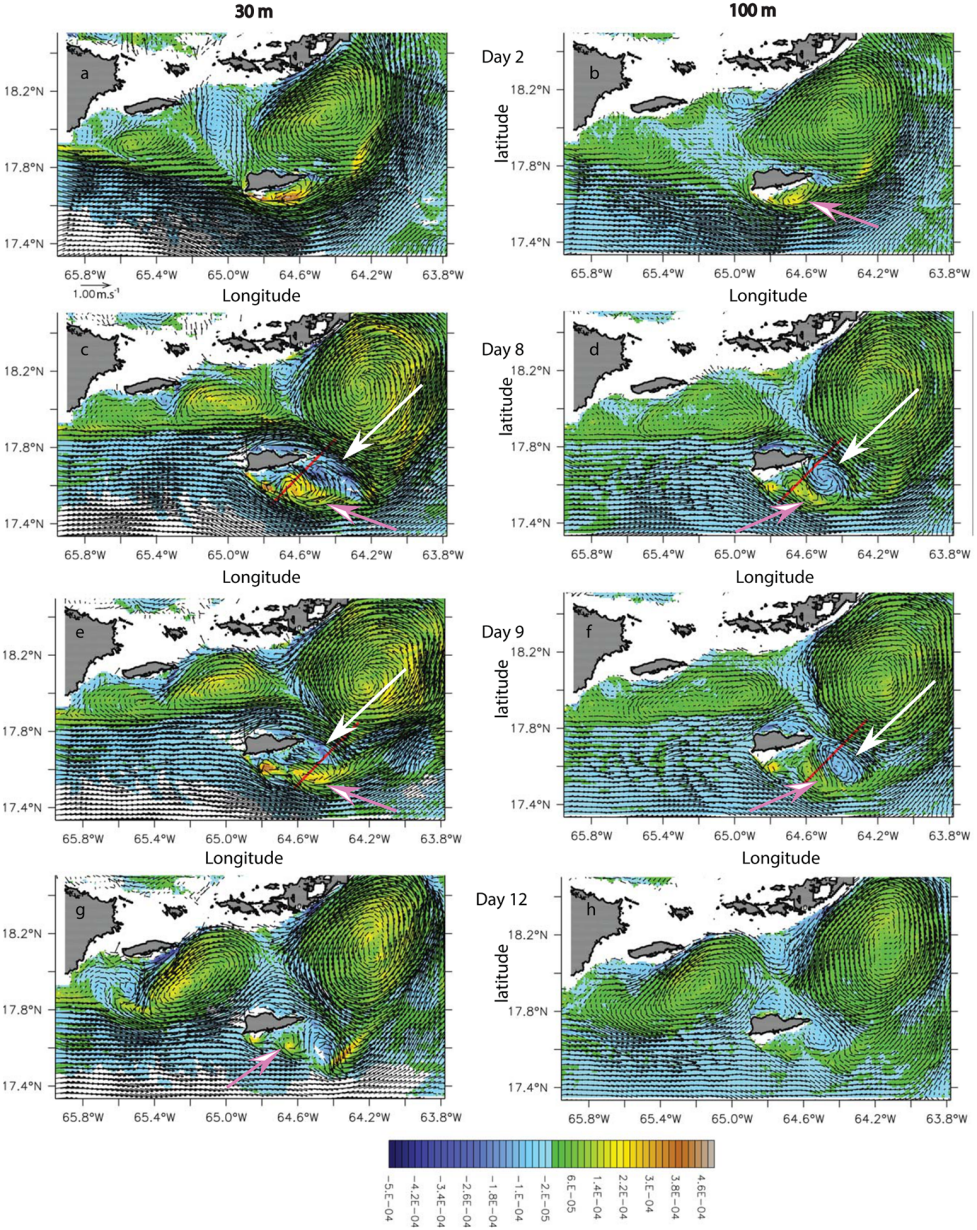
Potential vorticity anomaly (PVA) (s^-1^) maps overlaid with current vector (m.s^-1^) of the wake south of St. Croix in model year 2008 during the month of August for days 2, 8, 9 and 12. Left (right) column shows the vorticity at 30 (100)-m. Pink (white) arrows show cyclones (anticyclones). Red lines show the cross section locations used in Fig 12.

**Fig 12:**
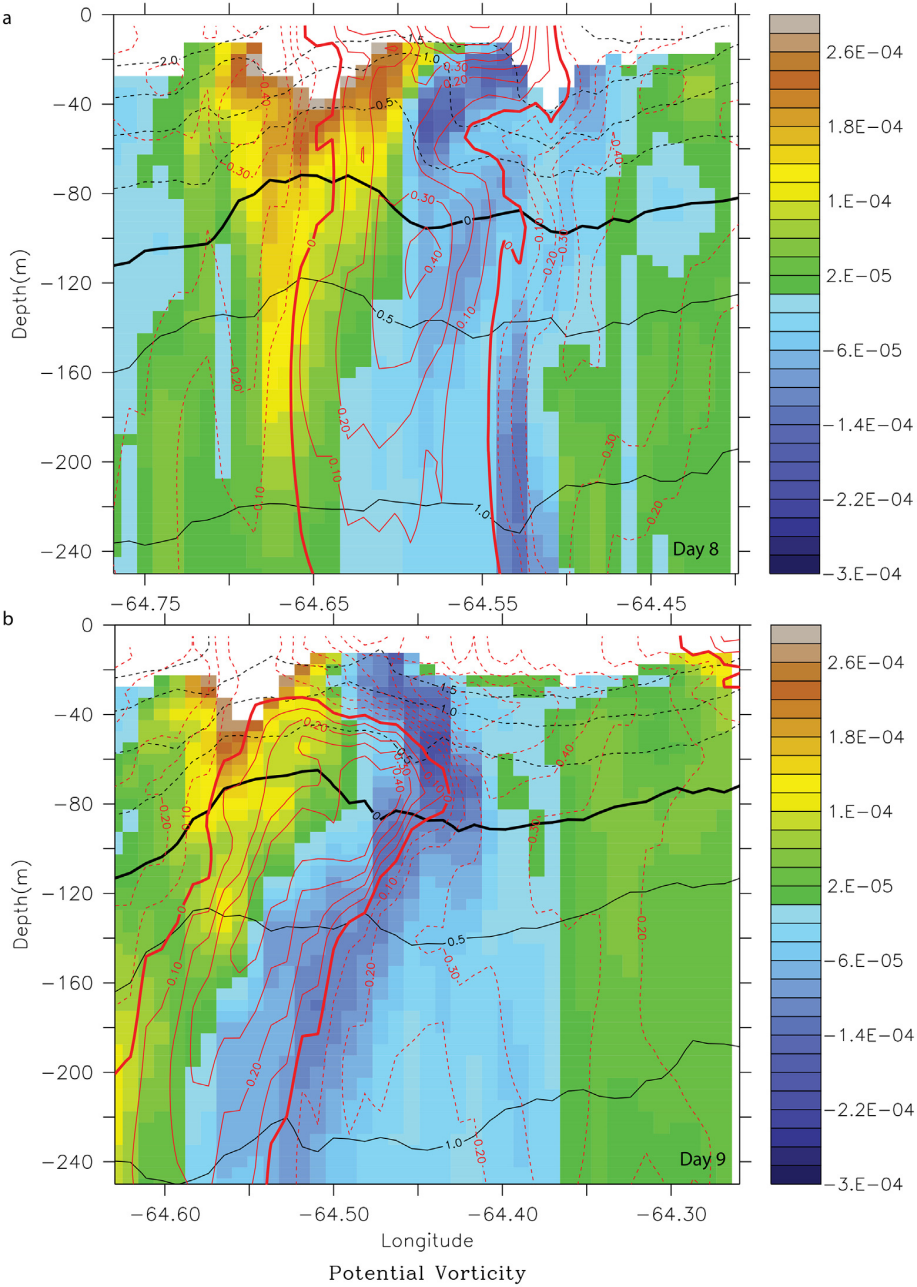
Potential vorticity anomaly (PVA) (s^-1^) cross-section of the dipole formed south of St. Croix during model month August 2008, on days 8 (a) and 9 (b). The PVA sections are overlaid with zonal velocity contours. Yellow-brown PVA shows the cyclone and blue-purple PVA shows the anticyclone.

In model month August 2008, the incident flow on St. Croix was eastward but deflected southward by the bottom topography on the southwest corner (Fig 11, right column). The incoming flow was relatively strong (0.5–1.0 m.s^-1^), and the wake flow regime consisted of short-lived cyclonic eddies (pink arrows) on the southern coast that traveled westward over the span of a week. They were surface-intensified with a deep signature (200 m; Fig 12). The northern branch of the incident flow also was forced southward and passed the island before forming an anticyclone (white arrows) that coupled with the former cyclone to form a dipole (Fig 11c–11f). As the cyclones shed from the southern cape, they were trapped between the anticyclone to the west and the southern branch of the incident flow to the east, which generated enough strain to tear the cyclones apart (Fig 11g and 11h). Cyclones generated in the lee of the southern promontory were either formed in the lee of the cape (Fig 11a and 11b; Day 2) with an approximate incoming velocity at depth of 1 m.s^-1^ or at the tip of the cape with an approximate velocity of 0.5 m.s^-1^ (Fig 11c–11h, Day 8–12). Because of the anticyclones that formed east of the cyclones, the latter were retained in the vicinity of the island. In this wake regime, the presence of bottom topography also played a critical role on the formation of wake eddies.

### Incident seasonal flow regimes

**Fig 13:**
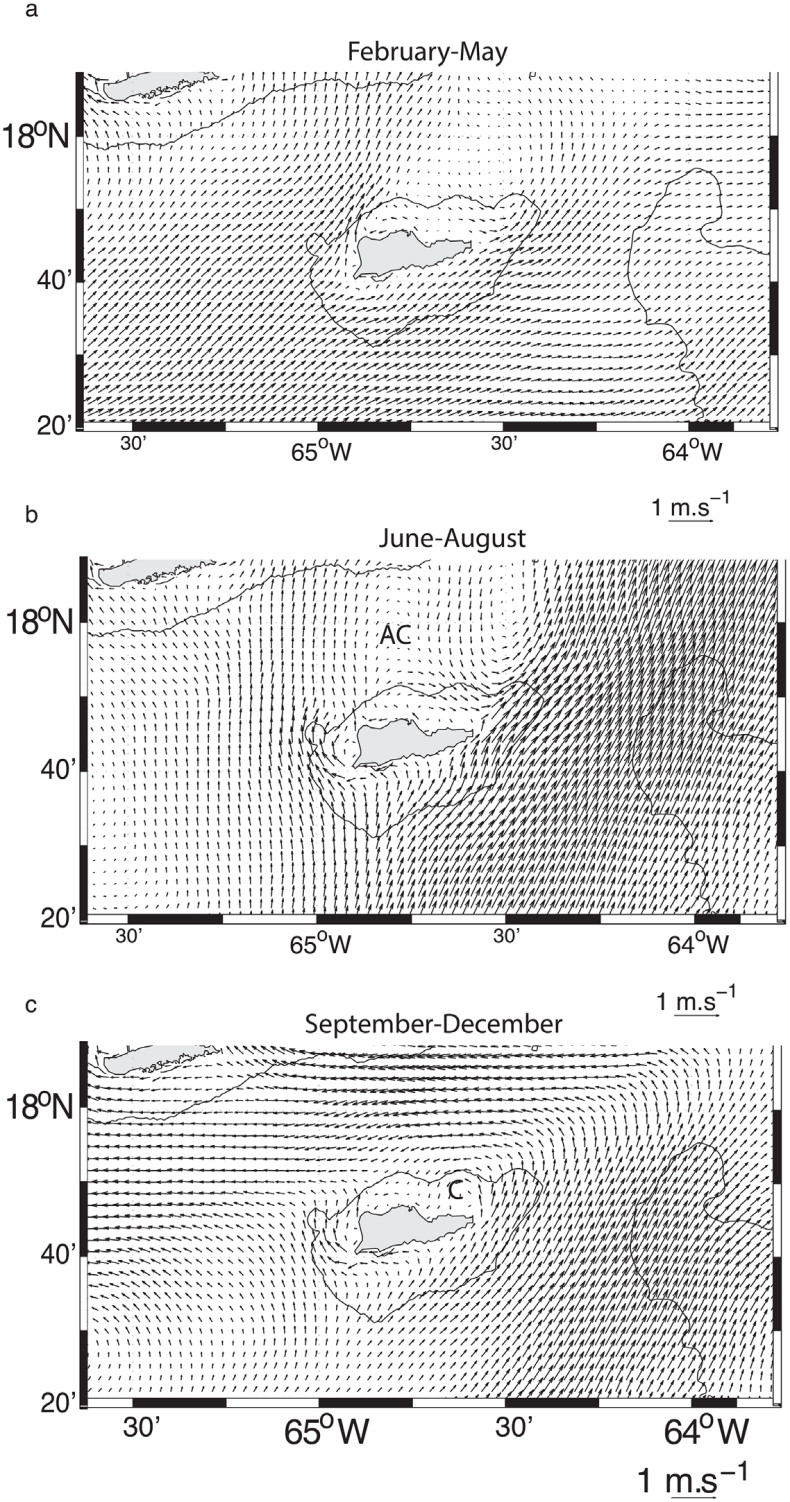
Three-year mean surface flow field in the first child grid obtained from the 2007–2009 model years. (a) Mean for the time period February-May. (b) Mean for the time period June-August. AC stands for “anticyclone”. (c) Mean for the time period September-December. C stands for “cyclone”

In sub-section 3.1, we showed that the St. Croix incident flow is driven by the riverine water-induced turbulent flow field, which contributes to a seasonal change in the dynamical state of the eastern Caribbean Sea [12]. Therefore the wake flow regimes may follow this seasonality, which we describe here, based on a three-year mean (2007–2009) of the model flow field (Fig 13). From February to June, the mean incident flow consists of a weak northeastward flow with a wake zone on the northeast of St Croix (Fig 13a). When the riverine water starts entering the eastern Caribbean Sea in July, the incident flow shifts more northward and is likely to generate an anticyclonic wake flow north of St. Croix as the velocity front remains east of the island (Fig 13b). In September-December, the incident flow remains northeastward to the east of the island but northward to the west of the island. The flow north of the island is westward, favoring a cyclone-dominated wake north of St. Croix (Fig 13c). This cycle should repeat every year as it is driven by the riverine water cycle shown in Hu et al. [11], but the timing of the plume’s arrival could alter it.

### Modeled larval dispersal of *T*. *bifasciatum*

The previous wake flow analysis revealed the existence of several types of wake between June and November that are characterized by long-lived coherent structures and/or short-lived shed cyclones. The wake zone is associated with a wind-driven upwelling that contributes to offshore transport at the surface and to shoreward transport below 20 m (Figs 4 and 8). Surface waters are fed with nutrient-rich waters because the nutricline lies on top of the SMW [32]. Offspring of reef fishes spawned in these areas have multiple opportunities to be transported offshore near the surface or inshore via deeper onshore flow on time scales of days, as well as to remain trapped for up to three weeks (eddy lifetime) or more if they interplay vertically within the flow field.

**Fig 14:**
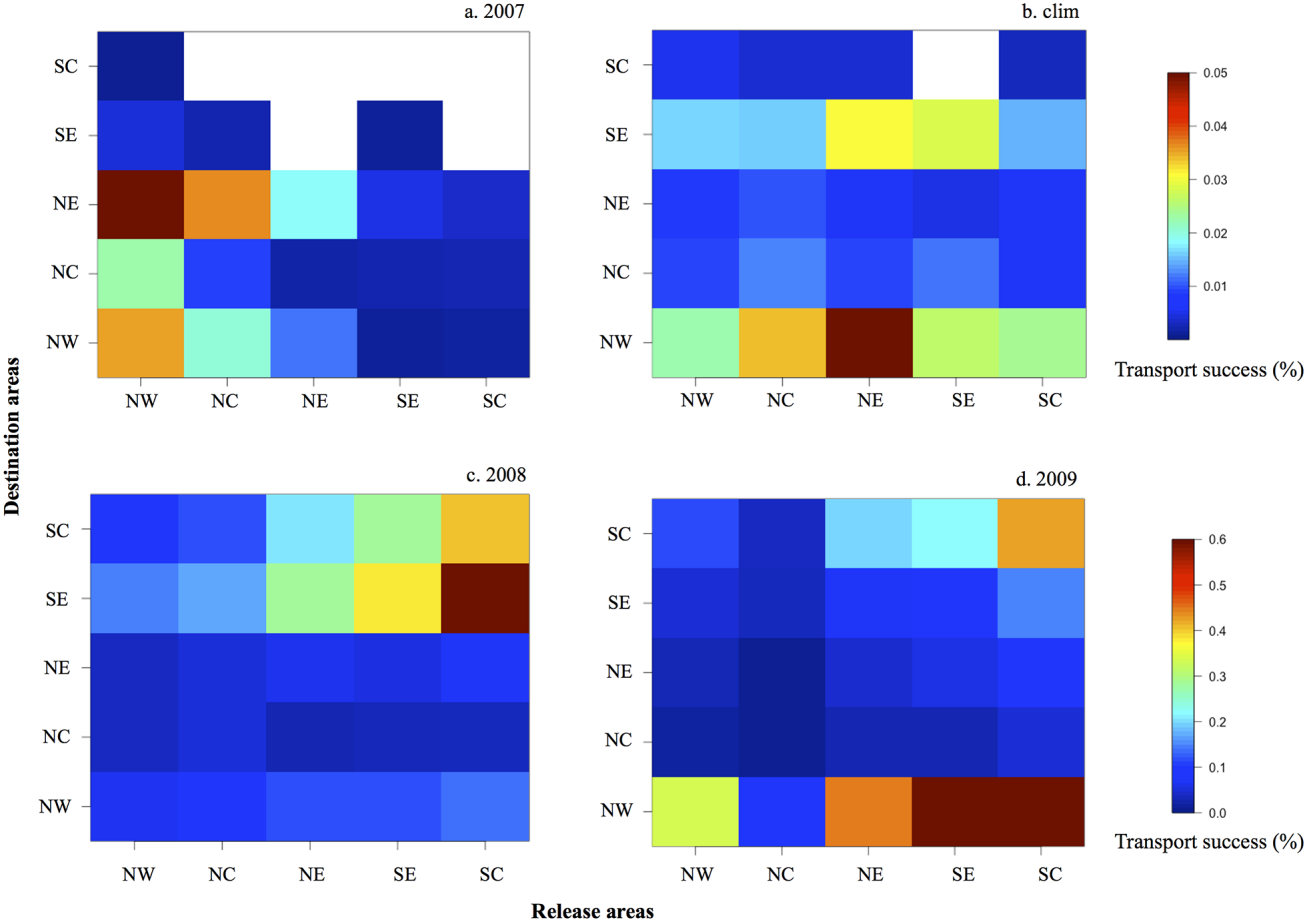
Connectivity matrices of simulated *T*. *bisfasciatum* larvae from five release areas: northwest NW, central north NC, northeast NE, southeast SE, and central south SC. For model year (a) 2007, (b) the climatological model, (c) 2008, and (d) 2009. “clim” = climatological.

The transport success of the settled larvae to the destination areas from June to September is represented as connectivity matrices in Fig 14. For model years 2007, 2009 and the climatological simulation, more larvae settled on the northern sites (Fig 14a, 14b and 14d; Table 1), whereas in 2008 we observed a higher transport success on the southern sites (Fig 14c; Table 1). On the northern shore, a gradient is observed from west to east: in 2007 the northeast (NE) site received more larvae, whereas in 2008, 2009, and the climatological simulation, the northwest (NW) site had the highest transport success of settled larvae. From the connectivity matrices, we can also observe the origin of the settled larvae for each model configuration. For model years 2007, 2009, and the climatological simulation, local retention in both the northern and southern shores is showed whereas in 2008 local retention occurred only in the southern shores. For model year 2009, larvae settled in the northern shore mainly came from the southern shore. Globally, the highest settlement rate on St. Croix occurred in model year 2008, followed by 2009 and the climatological year. In model year 2007, the larval settlement rate was the lowest.

**Table 1:** Percentage of the larvae recruiting on the northern and southern shore for the ROMS model configurations used (2007, 2008, 2009, and climatological). “clim” = climatological model.

	2007	2008	2009	clim
Northern Shore	98.5	18.1	72.6	72
Southern Shore	1.5	81.9	27.4	28

**Fig 15:**
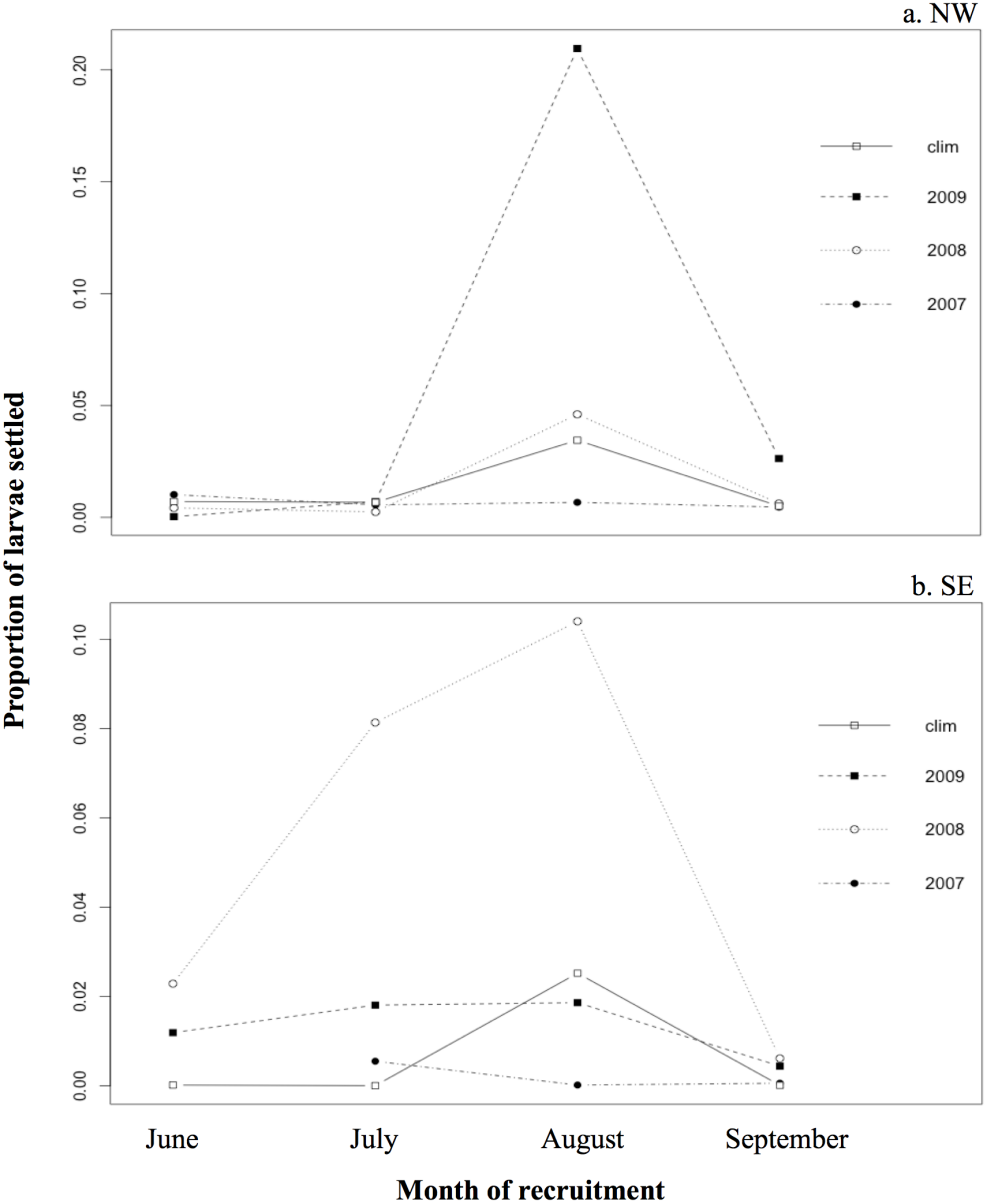
Proportion of larvae settled in (a) the northwest site (NW), and in (b) the southeast site (SE) in June, July, August, and September of model year 2007, 2008, 2009, and the climatological model. “clim” = climatological.

We also analyzed the monthly settlement of larvae on the northwest (NW) site and on the southeast (SE) site for each model year (Fig 15a and 15b, respectively). The greatest settlement on the northwest site occurred in August 2009, followed by August 2008 and the climatological year (Fig 15a). In June, July, and September, few larvae were transported to the northwest site regardless of the model year. At the southeast site, the proportion of larvae transported to the destination areas increased from June to August and decreased in September (Fig 15b). This is especially the case in 2008, but also in 2009 and the climatological year.

From our simulations of *T*. *bifasciatum* larval transport in St. Croix, we observed spatial and seasonal variability in recruitment. For all model years, our results showed distinct settlement patterns between northern and southern shores, with more larvae settled on the northern shore except in 2008 (Fig 14c). In 2008, 2009, and the climatological year, an eastward gradient of settlement was obtained on the northern shore; in 2007 and the climatological year, a westward gradient of settlement was obtained on the southern shore (Fig 14). Similar gradients have been seen in field studies [13, 14]. The northwestern shore (NW) is the highest recruitment site overall. Both the NW and the eastern south shore (SE) were shown by Hamilton et al. [14] to be the sites with highest recruitments in average as simulated during the climatological year. The across-site inter-annual variability that we saw with lower recruitment at NW than at SE in model year 2007 was also observed by Hamilton et al. [14]. However, in all years the larval settlement observed by Hamilton et al. [14] at SE was always the highest on the southern shore, whereas it was variable in our simulation (Fig 14).

## Discussion

### Wake flow dynamics and vorticity source

Following Batchelor [40], when a horizontal flow with uniform density encounters an obstacle in a non-rotating framework, a viscous horizontal boundary layer is formed along the solid boundary where vertical vorticity, ζ, is generated. As the flow passes the obstacle, it accelerates and the pressure decreases according to Bernoulli's law. An adverse pressure gradient may develop at the downstream edge of the boundary layer that can produce a flow reversal and detachment of the boundary layer from the solid wall, forming eddies that can be entrained in the wake. In their laboratory experiments, Batchelor [40] and Gerrard [41] showed that a two-dimensional flow around a cylinder is well characterized by Re. If Re<1, flow separation does not occur, and the flow is symmetric upstream and downstream. If 1<Re<40, a laminar separation occurs, and two steady vortices of opposite signs are formed downstream of the obstacle. At moderate Reynolds numbers, 40<Re<10^3^, the steady vortices are replaced by a periodic von Kármán vortex sheet. For Re>10^3^, the separated flow becomes increasingly turbulent and temporally irregular. When both rotation and stratification are present, the wake regimes are different, and in the presence of an extremely large Re value, oceanic and atmospheric wakes can significantly differ from homogeneous, non-rotating wakes with relative low Re values [42].

Wake dynamics are mostly driven by the production of vorticity by either bottom friction in the case of shallow waters or lateral boundary layers in the presence of deep waters surrounding an island [43]. In the case of a circular island surrounded by deep waters, Dong et al. [34] found three types of instability: centrifugal, barotropic, and baroclinic. The wake features were shown to be dependent on three non-dimensional parameters: Re, the Rossby number (Ro), and the Burger number (Bu). The dependence on Re was similar to Batchelor’s [40] classical wake, but for large Re, coherent eddies were still present in the island wake. Both anticyclonic and cyclonic eddies were formed in the wake. However, cyclonic eddies were more stable than anticyclones, which broke into several smaller anticyclones because of centrifugal instability. Decreasing Bu caused the diameter of cyclonic eddies to contract from island width to the baroclinic deformation radius, and the eddy generation process shifted from barotropic to baroclinic instability. For small Ro values, the wake dynamics were symmetric with respect to cyclonic and anticyclonic eddies. At intermediate Ro and Bu values, the anticyclonic eddies were increasingly more robust than cyclonic ones as Ro/Bu increased, but for large Re and Ro values, centrifugal instability weakened the anticyclonic eddies while cyclonic eddies remained coherent. The wake vertical structure also showed asymmetry between anticyclones, which were subsurface-intensified, and cyclones, which were surface-intensified.

With a non-circular island, the island’s tips or corners may act as a cape and modify the wake dynamics and vorticity-generation mechanisms described by Dong et al. [34]. In this case, several flow regimes were shown to be controlled by the equivalent Reynolds number Re_*f*_ [44], defined by the ratio between advection and bottom friction terms, although mostly in shallow water cases when bottom friction terms are non-negligible [45–47]. For very small Re_*f*_, the flow tends to follow the cape without separating. As Re_*f*_ increases, the flow regime evolves from laminar separation with an “attached” stationary eddy in the lee of the cape to eddy shedding with increased turbulence. Similar to the island wake flow regimes, other non-dimensional parameters (e.g., Ro and Bu) that characterize the effect of rotation and stratification were shown to play a role in the flow regime [48].

Therefore, the wake or leeward flow of St. Croix could be seen as the result of the combined effect of wake and cape dynamics, where the topographic control varies accordingly. In the presence of bottom topography forcing, eddies are stronger and live longer, while in the presence of lateral friction they are weaker.

### Larval settlement patterns

The study of the larval settlement seasonality in NW and SE sites revealed a monthly variability at both sites (Fig 15). August was the period of higher larval recruitment at the NW site (Fig 15a), which was also observed by Hamilton et al. [14]. However, Hamilton et al. [14] found an increase of *T*. *bifasciatum* recruitment in September at SE site, which was not observed in the model. Swearer et al. [49] studied the origin of *T*. *bifasciatum* larvae at different sites on northern and southern shores of St. Croix, finding that the recruits observed on the southern shore in autumn (September and October) originated from remote populations east of St. Croix. This could explain the difference with our model, which only considered larvae originating from St. Croix.

### Wake flow regime and recruitment variability

In agreement with Hamilton et al. [14], our multiyear simulation of recruitment showed a significant consistency in the recruitment patterns at the island scale and at the annual scale. The two studies show some variability, however, at the regional scale (shore-wide scale), which in our model can only be driven by oceanic variability because all other biotic factors in the biological model were kept the same. We therefore analyzed the wake regime and the subsequent recruitment spatial structure. In model year 2007, the wake consisted of an anticyclone on the north side of St. Croix with a cyclone-shedding regime on the east side. This wake flow seems to limit recruitment as shown by the connectivity matrix in Fig 14a. In 2008 the wake flow was on the southern side of St. Croix (Fig 11) and the highest recruitment site was on the southern shores (Fig 14c). In 2009 the wake flow was cyclonic on the north side of St. Croix (not shown) and recruitment results show that the northwestern cell was the most efficient at retaining larvae (Fig 14d). It also showed a gradient in recruitment on the southern shores. During the climatological year, the wake flow was also cyclonic and in some instances identical to Harlan et al. [15] HF radar observations. It also led to the highest recruitment on the northern shores (Fig 14b) and a gradient in recruitment on the southern shores. The cyclonic wake flow seems to be the type of wake driving most observed shore-wide gradients on both the north and south shores.

### Origin of wake flow variability

**Fig 16:**
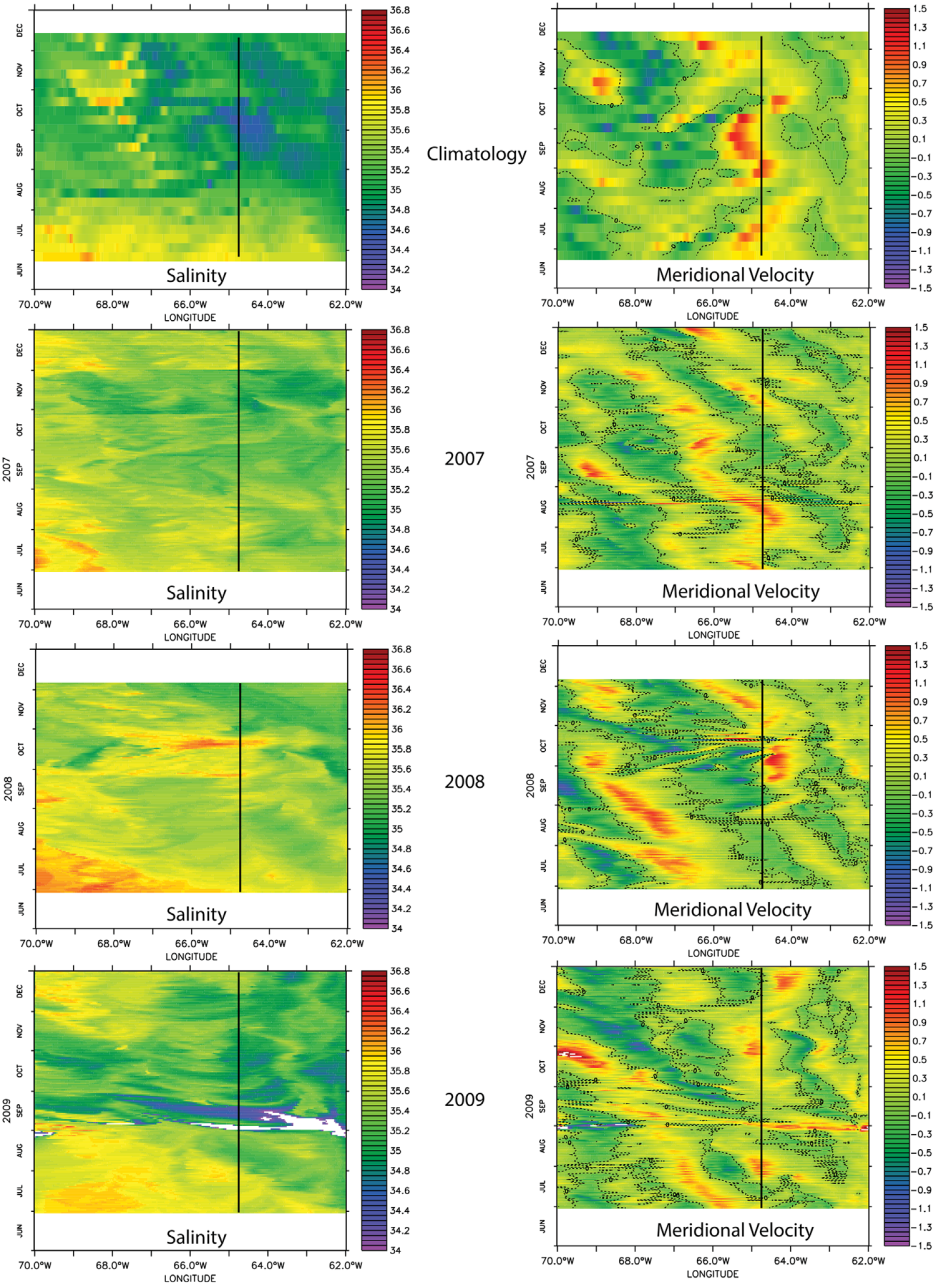
Phase salinity (left column) and meridional velocity (right column) diagrams in longitude and time (days) space for each of the ocean model years at 17°N.

Because the wake flow around St. Croix is driven by the transport of riverine water across the eastern Caribbean Basin [11, 12], we compared the incident circulation cell meridional velocities with the timing of passage of the riverine water plume south of St. Croix at the 17°N line, as shown in Fig 16. The timing of interest is July and August, when recruitment is the highest at all locations (Fig 15). During the climatological year, August is characterized by the beginning of the salinity decrease all across the model longitude. The velocity diagram shows in July and in late August a mesoscale cyclonic cell with northward incoming flow on St. Croix and southward flow to the west. This pattern suggests a cyclonic circulation, which is consistent with the cyclonic wake flow. In model year 2007, the salinity front is not as marked as in the climatological year, and the incident flow is also northward with southward flow east of St. Croix in July and both east and west of the island in August. These currents were associated with a wake dominated by an anticyclonic circulation north of St. Croix. The riverine water northward extension appeared to be limited. In model year 2008, the salinity change was weaker and later than in model year 2007 (August vs. July), and the incident flow was southward, which explains the wake south of St. Croix. In model year 2009, the salinity change at the St. Croix longitude is of similar amplitude as during the climatological year. Similarly, the incident flow was northward on the east side of St. Croix and southward on the west side in late July and early August as the riverine water plume arrived. This flow pattern created a cyclonic wake flow pattern on the northwest shores of St. Croix with substantial recruitment in that area as shown in Fig 15a.

This analysis suggests that the northward penetration of the riverine water is directly correlated to the type of wake around St. Croix, which determines the recruitment level spatial pattern around the island. However, the seasonal arrival of the riverine water seems to be the main consistency driver of the island wake, which may not exist in boreal winter and spring. Incident current on St. Croix during this time could be very weak because of the absence of significant salinity gradients and the fact that currents during those months tend to intensify west of St. Croix [12].

## Conclusions

We used a biophysical numerical model, Ichthyop, coupled with an ocean numerical model, ROMS, to simulate the larval transport of *T*. *bifasciatum*. Our first step was to validate the ocean model. We showed that the riverine water from the Amazon and Orinoco rivers was transported northward in the eastern Caribbean, as observed in SeaWiFS *Chl-a* images. We also confirmed the wake flow patterns north of St. Croix when dominated by a cyclonic circulation by comparing snapshots of the flow between the model and observations by Harlan et al. [15]. This step confirmed that the model is able to reproduce the main features of the circulation around St. Croix at the mesoscale and sub-mesoscale. Our second step was to validate the biophysical model, which showed the same spatial recruitment levels patterns as observed by Swearer et al. [49]. We then used a multiyear simulation to understand the wake dynamics and structure, as well as the correlation between the wake flow and the recruitment pattern. We found a great diversity of wake flows, including long-lasting (up to 20 days) attached eddies on the northern side of St. Croix and an eddy cannon at the eastern end of St Croix. The swift sub-mesoscale cyclonic eddies vorticity reached about -1.5*f* soon after detachment and rapidly decreased after three days (Fig 9). Similarly, wake cyclonic sub-mesoscale eddies generated in the lee of St. Croix’s southwest corner were also superinertial as shown in Fig 12. Interestingly, the anticyclone that they coupled with was also superinertial for a few days (Fig 12). This type of superinertial eddy field has also been observed in the lee of the Hawaiian Islands by Chavanne et al. [50], suggesting that island wake regions are favorable to the formation of such superinertial submesocale eddies. We found that cyclonic wake flows to the north of St. Croix were more likely to generate recruitment of more than 70% of the larvae on the northwest shore. In contrast, anticyclonic wake flow was likely to drastically limit the number of recruits, whereas southern wake would increase the recruitment levels on the windward shore. But unlike Swearer et al. [49] and Hamilton et al. [14], we did not find that the southeast shores were the highest recruitment zones in September, most likely because we did not account for dispersing recruits from the Windward Islands such as Saba, Saba Bank, or St. Eustatius, or other islands of the Lesser Antilles [49].

Finally, the multiyear study showed that recruitment was driven by wake flow around St. Croix at the annual scale, whereas wake flow type, which appears to control the recruitment level pattern at a seasonal scale, was driven by the northward penetration of riverine water plume. The further north the plume, the more likely the wake was to be cyclonic and on the northern shore, and if the salinity front was moved eastward and southward, the wake evolved from anticylonic north of St. Croix to cyclonic south of St. Croix. This correlation shows that the wake flow around St. Croix, and hence the recruitment pattern around the island, is driven by the fluxes of riverine water from the Amazon and Orinoco rivers in the Caribbean Sea. This correlation could also be of significance for other marine species around St. Croix, creating a modulation of recruitment levels and patterns due to the hydrological cycle of the Amazon and Orinoco rivers. Similar recruitment variability is then to be expected on the other shores of the Lesser Antilles.
